# Tailoring Electronic Structure of NbB_2_ Nanorods Through Selenium Incorporation for Synergistically Enhanced Hydrogen Evolution and Triboelectric Energy Harvesting

**DOI:** 10.1002/advs.77902

**Published:** 2026-09-27

**Authors:** Shalmali R Burse, Vikram Mahamiya, Ali Mohammadi, Swapnil R Patil, Harshitha B Tyagaraj, Gagankumar S K, Amal AlGhafri, Ebrahim Al Hajri, Nilesh R. Chodankar, Yun Suk Huh, Young‐Kyu Han

**Affiliations:** ^1^ Department of Energy and Material Engineering Dongguk University Seoul Republic of Korea; ^2^ Center for Interdisciplinary Research, Asian Consortium on Computational Material Science‐Global Research Centre (ACCMS‐GRC) SRM University‐AP Amaravati Andhra Pradesh India; ^3^ Department of Physics SRM University‐AP Amaravati Andhra Pradesh India; ^4^ Department of Biological Sciences and Bioengineering, Nanobio High‐Tech Materials Research Center Inha University Incheon Republic of Korea; ^5^ Rabdan Academy Abu Dhabi United Arab Emirates; ^6^ Mechanical and Nuclear Engineering Department Khalifa University of Science and Technology Abu Dhabi United Arab Emirates

**Keywords:** bifunctional electrocatalyst, hydrogen evolution reaction, microwave‐assisted doping, molten salt synthesis, niobium boride, overall water splitting, triboelectric nanogenerator

## Abstract

The sluggish hydrogen evolution reaction (HER) kinetics of non‐noble‐metal catalysts remain a major challenge for efficient hydrogen production. Herein, selenium‐doped niobium diboride (Se‐NbB_2_) nanorods were synthesized via a molten‐salt‐assisted route followed by microwave‐assisted treatment, yielding a catalyst with higher conductivity and superior electrocatalytic performance. HER activity reveals that Se‐NbB_2_ exhibits excellent performance, requiring an overpotential of only 147 mV to achieve 10 mA cm^−^^2^ and maintaining stable operation for more than 6 days under alkaline conditions. Furthermore, the catalyst demonstrates efficient bifunctional water‐splitting performance, delivering 10 mA cm^−^^2^ at a cell voltage of 1.79 V. First‐principles calculations reveal that Se incorporation modulates the electronic structure of NbB_2_ by shifting the Nb d‐band center upward and improving electrical conductivity, thereby optimizing hydrogen adsorption free energy toward thermoneutral conditions and accelerating HER kinetics. Beyond electrocatalysis, Se‐NbB_2_ was employed as an electrode material in a triboelectric nanogenerator (TENG), generating a maximum output voltage of 703 V and a current of 33 µA. The enhanced catalytic and triboelectric performances are attributed to improved conductivity and charge‐transport characteristics induced by selenium incorporation. This work highlights heteroatom‐mediated electronic‐structure engineering as an effective strategy for developing multifunctional transition‐metal boride materials for sustainable energy conversion, hydrogen generation, and energy‐harvesting technologies.

## Graphical abstract

Selenium incorporation modulates the electronic structure of niobium diboride (Se‐NbB_2_) nanorods, enhancing the active site density and resulting in superior hydrogen evolution performance for sustainable energy applications.

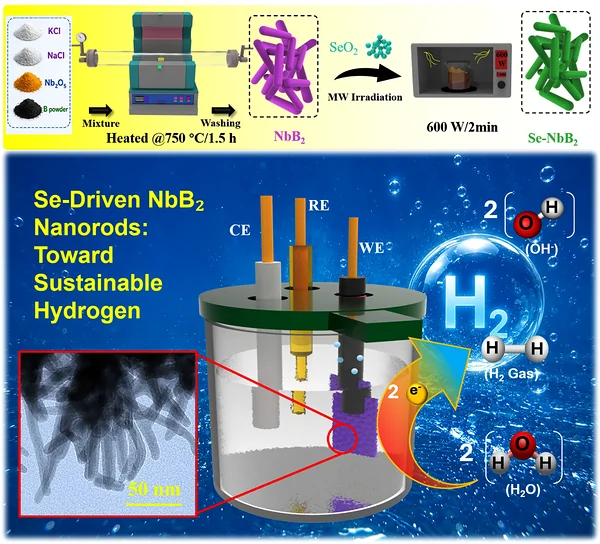

## 1 Introduction

Given the environmental problems caused by fossil fuel consumption, hydrogen (H_2_) is considered an alternative green energy source with zero carbon emissions [1, 2]. Among all hydrogen production approaches, electrochemical water splitting is an appealing and promising method due to its low cost and high efficiency [3, 4]. Overall, reducing the electrochemical overpotential and enhancing energy conversion efficiency are primary goals for the hydrogen evolution reaction (HER), and these goals are achieved by developing an efficient catalyst [5, 6]. The noble metals, particularly platinum, are widely considered state‐of‐the‐art catalysts for the HER owing to their superior intrinsic activity. Although platinum‐based catalysts exhibit low overpotential and exceptional efficiency for the HER, their high cost and scarcity hinder large‐scale application [7]. Therefore, the development of cost‐effective, earth‐abundant alternatives to replace platinum has become a critical focus in advancing economically viable water splitting technologies. In this context, a wide range of non‐noble metal‐based catalysts, including oxides [8], hydroxides [9], sulfides [10], selenides [11], nitrides [12], phosphides [13], and borides [14] have been extensively explored as promising substitutes for noble metals due to their low cost and natural abundance [15]. Among these non‐noble metal catalysts, transition‐metal borides (TMBs) have recently attracted considerable attention due to their outstanding mechanical robustness, corrosion resistance, and high thermal and electrical conductivity [16]. These properties arise from their unique bonding characteristics, including metal−metal, metal−boron, and boron−boron interactions [17, 18]. This multicenter boron bonding nature causes electronic orbital rearrangement within the lattice, which enhances water adsorption and promotes interactions with hydroxyl groups, thereby boosting catalytic activity [19, 20]. For instance, Chen et al. confirmed that α‐MoB_2_ can efficiently work as an HER catalyst at high current densities. The density and activity of hydrogen adsorption sites on the theoretical model α‐MoB_2_ surface were higher than those on the Pt surface [21]. Among TMBs, NbB_2_ is particularly attractive for the HER because its AlB_2_‐type layered lattice combines metallic conductivity from Nb−Nb networks with tunable H* adsorption enabled by Nb 4d−B 2p hybridization and the multiple accessible oxidation states of Nb [22, 23, 24, 25]. Therefore, the development of advanced synthesis strategies for NbB_2_ that overcome existing fabrication challenges is crucial to accelerating its practical application in water splitting technologies. In addition, tuning the electronic structure of catalysts through surface engineering or doping is an effective strategy to enhance water splitting activity. For example, Khossossi et al. demonstrated through theoretical studies that Fe/Co doping on NbB_2_ surfaces can generate highly active HER sites [26].

Among various strategies, heteroatom doping, particularly with chalcogen elements (S, Se, and Te), has proven effective in tuning the electronic structure and enhancing the catalytic performance of a wide range of catalysts [27]. This approach introduces lattice defects and additional active sites without disrupting the host structure, thereby significantly boosting overall water splitting activity for clean H_2_ production [11, 28]. Among the chalcogens, Se is particularly attractive for modulating transition metal borides. Compared with sulfur, Se has substantially higher electrical conductivity (semiconducting Se:10^−^^3^ S m^−^^1^ vs. insulating S:10^−^^15^ S m^−^^1^) and a slightly lower Pauling electronegativity (Se 2.55 vs. S 2.58), so metal−Se bonds are more covalent and metallic in character than the corresponding metal−S bonds, which promotes electron redistribution and accelerates interfacial charge transfer. In addition, the larger atomic radius of Se induces moderate lattice expansion upon substitution, generating defect‐rich active sites, while the relatively weak Se−H interaction is known to lower the hydrogen desorption barrier, thereby accelerating HER kinetics [29, 30]. Moreover, selenides exhibit lower H_2_ desorption energy, which is beneficial for accelerating HER kinetics [31]. Previous studies have shown that Se doping enhances the alkaline HER performance of CoP by atomic‐level electronic structure modulation and optimizing the adsorption behavior of reaction intermediates at the atomic level [32]. Similarly, Se incorporation into the Ni−Co hybrid system effectively boosts the electrocatalytic performance for overall water splitting by enhancing intrinsic activity, promoting interfacial charge transfer, and increasing the density of active sites [33]. Nb 4d states dominate the HER‐relevant frontier orbitals of NbB_2_, and Se incorporation can shift the Nb d‐band center upward and bring ΔG_H_ close to thermoneutral [34, 35]. The material conductivity and surface charge states have a direct influence not only on electrochemical catalysis but also on triboelectric nanogenerators (TENG) [36]. Thus, employing Se‐NbB_2_ across both HER and TENG platforms highlights a unified approach to validating broadly applicable material design principles.

In this work, Se‐doped NbB_2_ was synthesized via an innovative molten salt method followed by microwave (MW) treatment, yielding interconnected NbB_2_ nanorods with rapid Se incorporation. Importantly, the molten salt and MW approaches serve distinct but complementary purposes. The molten salt method provides a highly reactive and diffusion‐enhanced medium that promotes intimate contact between the precursors, facilitates mass transport, and favors the formation of highly crystalline NbB_2_ at a lower processing temperature than conventional solid‐state synthesis. Subsequently, MW irradiation enables rapid and efficient Se incorporation through accelerated heating and diffusion, avoiding the prolonged high‐temperature treatments typically required for heteroatom doping. In previously reported boride systems, heteroatom doping has generally required prolonged high‐temperature treatment and time. For instance, La doping in MoBx was achieved using a molten salt method at 800 °C for 4 h, whereas S‐doped ReB_2_ was prepared by thermal treatment at 1000°C for 1 h [37, 38]. Such conventional high‐temperature processes require extended reaction times and substantial thermal energy. In contrast, the present Se‐doping process is completed within only 2 min at 100–110°C using MW irradiation, offering a significant reduction in both processing time and energy consumption.

Although heteroatom engineering of metal borides has been extensively investigated, previous studies mainly depend on conventional, time‐intensive doping and often lack comprehensive mechanistic analysis. Furthermore, studies on Se‐modified metal borides remain relatively limited compared with other heteroatom‐doping strategies, such as S, P, and N doping. The present work employs an ultrafast MW‐assisted Se‐doping strategy, providing a time‐ and energy‐efficient alternative for Se incorporation into NbB_2_. Furthermore, combining ultrafast Se doping with in situ Raman spectroscopy and density functional theory (DFT) calculations provides comprehensive insights into the structural and electronic origins of the enhanced HER performance. More importantly, the Se‐NbB_2_ prepared with this combined approach effectively modulates its electronic structure and surface chemistry, thereby increasing the density of catalytically active sites and enhancing its intrinsic HER activity. A comparison of the present work with previously reported heteroatom‐modified boride systems is provided in Table S1. The optimized Se‐NbB_2_ exhibits HER activity that needs only 147 mV@10 mAcm^−^^2^ in 1 M KOH electrolyte, which is significantly lower than NbB_2_, (287 mV @10 mAcm^−^^2^) and remarkably long term stability. Moreover, Se‐NbB_2_ shows outstanding output voltage and current as an electrode in TENG devices. The incorporation of Se into the NbB_2_ structure can significantly enhance catalytic activity and energy conversion efficiency in TENG devices.

## 2 Experimental

### 2.1 Materials

Niobium oxide (Nb_2_O_5_), boron powder, sodium chloride (NaCl), potassium chloride (KCl), selenium dioxide (SeO_2_), hydrazine hydrate, potassium hydroxide (KOH), and Nafion 5 wt.% were purchased from Sigma–Aldrich and used without further purification. Further, ultrapure water was used to prepare all the solutions throughout the experiments.

### 2.2 Synthesis of NbB_2_

NbB_2_ was synthesized via a molten‐salt‐assisted reduction process. In a typical procedure, 0.14 g of Nb_2_O_5_, 0.04 g of boron powder, 3 g of NaCl, and 3 g of KCl (molar ratio is 1:1) were thoroughly ground for 30 min to obtain a homogeneous mixture. The mixed powders were then transferred into a porcelain crucible and heated in a tube furnace at 750°C for 1.5 h under a continuous flow of argon. After naturally cooling down to room temperature, the resulting product was dissolved in hot deionized water and subsequently washed with ethanol/water to remove residual molten salts and byproducts. The obtained black powder was collected and dried under vacuum at 70°C overnight.

### 2.3 Synthesis of Se‐NbB_2_

Selenium‐doped niobium diboride (Se‐NbB_2_) was prepared by using a microwave (MW)‐assisted reduction method. Briefly, 200 mg of NbB_2_ powder was dispersed in 50 mL of deionized water under stirring, followed by the addition of 3.87, 11.6, 19.4, and 38.8 mg of SeO_2_ powder to prepare different doping contents of Se in NbB_2_. We named these samples as Se1‐NbB_2_, Se3‐NbB_2_, Se5‐NbB_2_, and Se10‐NbB_2_, respectively. Subsequently, 30 µL of hydrazine hydrate was slowly introduced into the suspension as a reducing agent. The mixture was then subjected to microwave irradiation with a constant power of 600 W for 2 min. After completion of the reaction, the product was collected, washed, and dried under vacuum at 70 °C overnight.

### 2.4 Characterization

Field emission scanning electron microscopy (FE‐SEM) images were obtained using an HR‐SEM (HITACHI S‐4800, Japan). A high‐resolution transmission electron microscope (HR‐TEM) and an energy‐dispersive X‐ray spectroscope (EDS) were used on an HR‐TEM, JEM‐2100F, JEOL, Japan. X‐ray diffraction (XRD, X'Pert‐PRO MRD, Philips, The Netherlands) using Cu Kα irradiation and X‐ray photoelectron microscopy (XPS, Thermo Fisher Scientific, K‐alpha, USA) were used to examine the phase structure and surface chemistry. Raman spectroscopy was performed using a laser Raman spectrometer (Raman, FEX, NOST, Republic of Korea) with a 532 nm excitation source. Fourier‐transform infrared (FT‐IR) spectroscopy (JASCO FT/IR‐6600, Japan) was used to analyze the functional groups and bonding characteristics of the materials. The surface area was determined by Brunauer–Emmett–Teller (BET) measurements, and density functional theory (DFT) calculations were performed using the Vienna Ab‐initio Simulation Package (VASP).

### 2.5 Preparation of Electrodes

The working electrode was prepared on the carbon cloth (CC). First, the CC was treated with a 10 mM HNO_3_ solution for 1 h and then cleaned with deionized (DI) water several times, followed by drying in a vacuum oven at 70°C for 2 h. 10 mg of selected catalyst powder was dispersed in 20 µL of 5 wt.% Nafion, 380 µL of isopropanol, and 600 µL of DI water. The mixture was sonicated for 30 min to form a homogeneous ink. Further, 150 µL of the catalyst ink was drop‐cast onto the CC substrate, covering an area of 1 × 1 cm. The drop‐casted CC was further dried in a vacuum oven at 70°C for 5 h. Commercial Pt/C or RuO_2_ electrodes were made in the same way.

### 2.6 Electrochemical Measurements

The electrochemical measurements were performed on a CHI 7089E electrochemical workstation in a three‐electrode system. 1.0 M KOH was used as an electrolyte. The catalyst powder was deposited on the carbon cloth used as a working electrode. The Pt plate and Hg/HgO were used as the counter and reference electrodes, respectively. Hg/HgO was calibrated before the measurements. All the potentials were without iR‐compensation and calibrated to the reversible hydrogen electrode (RHE) by the formula E_RHE_ = E_Hg/HgO_ + 0.098 + 0.059×pH. The HER/OER performance was evaluated by linear sweep voltammetry (LSV) with a scan rate of 5 mV s^−^^1^. The Tafel slopes were derived from the corresponding LSV curves. Electrochemical impedance spectroscopy (EIS) was conducted at varied voltages corresponding to the fixed current density of 10 mA/cm^2^ between 100 kHz and 0.1 Hz with an amplitude of 5 mV. To analyze the electrochemically active surface area of the catalyst using cyclic voltammetry (CV) curves in the potential range of 0.325‐0.175 V (vs. RHE, non‐Faradaic region), measurements were made under different scan rates of 20,40, 60, 80, 100, 120, 140, and 160 mV s^−^^1^ and were utilized to calculate double‐layer capacitance (*C_dl_*) values of different electrodes. Related to the C*_dl_* and anodic and cathodic currents calculated by the equation *∆j* = (*Ja*−*Jc*)/2. HER stability was measured by chronoamperometry over 30 h. Further, a two‐electrode cell was configured with the Se5‐NbB_2_ electrode as both anode and cathode. The electrochemically active surface area (ECSA) was obtained according to ECSA = $\frac{C_{dl}}{C_{s}},$ where Cs is surface capacitance = 40 µF cm^−^^2^. The long‐term stability of the catalyst was studied via chronopotentiometry measurements at different current densities of 10, 20, 60, 100, 140 mA/cm^2^. The note is that the reproducibility of the present system was ensured through LSV, and an overpotential difference of ±2 mV was allowed at the same current density.

### 2.7 In Situ Raman Measurement

In situ Raman measurements were conducted using a Raman spectrometer (RAON‐Spec, NOST, Republic of Korea) equipped with a 532 nm Nd:YAG laser operating at a power of 50 mW. An in situ electrochemical Raman cell with a volume of 30 mL was employed. The cell body was fabricated from polytetrafluoroethylene (PTFE) and custom‐manufactured by Dek Research Company. The Raman cell was fitted with a JGS2 optical quartz window (φ41 × 1 mm). A platinum wire electrode (φ0.5 × 37 mm) served as the counter electrode, while an Hg/HgO electrode was used as the reference electrode. The electrolyte consisted of 1 M KOH solution. Electrochemical measurements, including chronopotentiometry, were performed using a CHI 7089E electrochemical workstation.

### 2.8 Computational Details

We performed density functional theory (DFT) calculations using the Vienna Ab‐initio Simulation Package (VASP) employing the Perdew‐Burke‐Ernzerhof (PBE) exchange‐correlation functional. A plane‐wave energy cutoff of 520 eV was used throughout all calculations. The convergence criteria for electronic self‐consistency and ionic relaxation were set to 10^−^^6^ eV for total energy and 0.01 eV Å^−^^1^ for atomic forces, respectively. For modelling the NbB_2_ surface, a vacuum region of 20 Å was introduced along the surface normal to eliminate spurious interactions between periodic images. Brillouin zone sampling was performed using a Γ‐cantered k‐point grid of 6 × 6 × 1 for structural relaxations, while a denser grid of 8 × 8 × 1 was employed for self‐consistent field (SCF) calculations to ensure accurate electronic properties. Dispersion interactions were accounted for using the Grimme DFT‐D3 method. Additionally, dipole corrections were applied to remove artificial electrostatic interactions arising from the asymmetric slab geometry. The zero‐point energy (ZPE) corrections were computed for the adsorbed hydrogen species by considering vibrational contributions of the hydrogen atom, while the surface atoms were assumed to remain effectively fixed.

## 3 Results and Discussion

### 3.1 Morphological and Structural Analysis for Se‐NbB_2_ Nanorods

**FIGURE 1:**
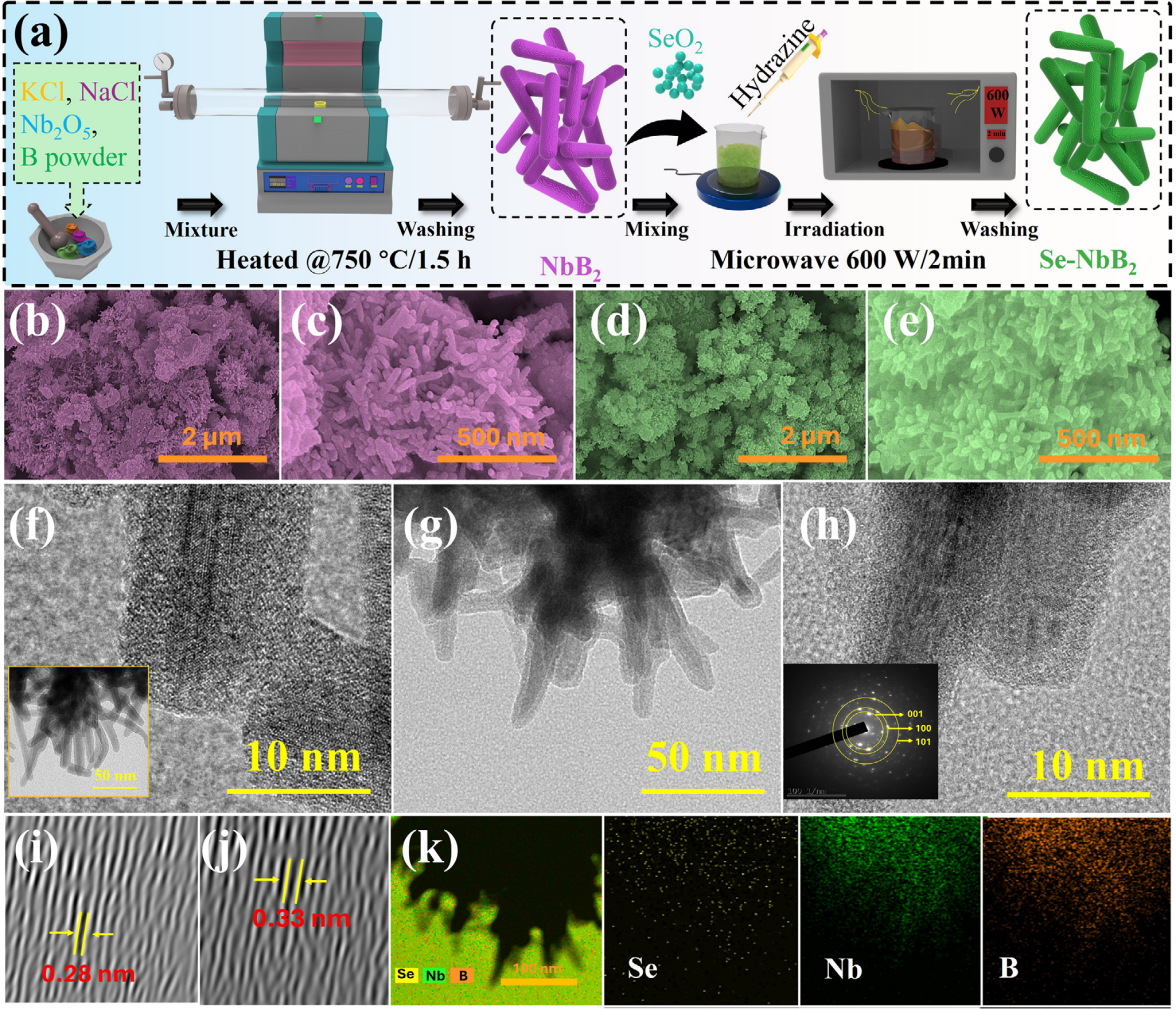
(a) Schematic of the synthesis process of NbB_2_ and Se‐NbB_2_. SEM images of (b‐c) pristine NbB_2_ and (d,e) Se‐NbB_2_. HR‐TEM images of (f and i) NbB_2_. (g, h, andj) Se‐NbB_2_. (k) EDX elemental mapping for Se5‐NbB_2_.

Figure 1a shows the schematic preparation steps for NbB_2_ and Se‐NbB_2_ via the facile molten salt method (KCl/NaCl) followed by rapid MW processing. The Nb_2_O_5_ and boron (B) powder were used as precursors for preparing NbB_2_. Using the facile molten salt method it can ensure the highly crystalline nature of NbB_2_. The NbB_2_ phase formation reaction was carried out under an inert atmosphere using Ar gas. The overall reaction can be described as follows:

(1) $$3Nb_{2}O_{5}+22B\to 6\mathrm{Nb}B_{2}+5B_{2}O_{3}$$

Under high‐temperature conditions, boron species may be partially lost via volatilization and dissolution, necessitating the use of excess boron to ensure the formation of stoichiometric and phase‐pure NbB_2_ [23]. The KCl/NaCl eutectic salt mixtures melt to form a liquid reaction medium, which promotes homogeneous nucleation and controlled crystal growth of NbB_2_, enabling the formation of a pure phase of NbB_2_ [39]. Previously, NbB_2_ was synthesized by solid‐state or hydrothermal routes using a 12–15 h process, yielding aggregated nanorods or nanoparticles and again requiring high‐pressure vessels [40]. Moreover, crystallization in the molten salt medium can create a dynamically evolving lattice environment with defect active sites, which are favorable for heteroatom incorporation (e.g., Se doping) into NbB_2_. Se1‐NbB_2_, Se3‐NbB_2_, Se5‐NbB_2_, and Se10‐NbB_2_ represent increasing nominal SeO_2_‐to‐NbB_2_ weight ratios based on precursor amounts (Section 2.3). These defects play a crucial role in modulating the electronic structure and generating catalytically active sites, thereby enhancing HER performance. Subsequently, Se is introduced via rapid MW‐assisted synthesis (600 W, 2 min), during which Se atoms are incorporated into the NbB_2_ lattice.

Most of the time, doping of heteroatoms or metals has been achieved either in a tube furnace with an inert atmosphere or via a hydrothermal process. Both methods are time‐ and energy‐consuming, and during doping, the nanostructure of the base materials often changes [41, 42]. MW‐assisted synthesis is a potential route to accelerate the doping reaction owing to its rapid, selective heating characteristics [43]. MW heating can deliver energy spontaneously to targeted components interacting with the reactants via an alternating electromagnetic field. A short irradiation time (2 min) was employed to ensure rapid reduction of SeO_2_ in hydrazine solution, while avoiding excessive heating, which can lead to Se loss or structural degradation of NbB_2_ [44]. Although the reaction temperature (100–110°C) during MW irradiation is relatively low, MW‐assisted heating generates localized hotspots and rapid interfacial heating. As a result, the reducing power of hydrazine is sufficient to reduce SeO_2_ and effectively dope Se into NbB_2_ [45, 46]. The MW process induces lattice distortion, defect generation, and modulation of the electronic structure, leading to the formation of Nb−Se/B−Se bonding [47]. As a result, Se doping effectively tailors the surface chemistry and electronic properties of NbB_2_, creating abundant catalytically active sites that favor enhanced water splitting reactions [48]. The detailed synthesis process for Se‐NbB_2_ is provided in Section 2.3. Initially, pristine NbB_2_ is synthesized at various reaction temperatures to optimize phase formation and morphology. The SEM image of NbB_2_ at different temperatures is shown in Figure S1a–d. At 650 °C, the NbB_2_ sample exhibits irregularly aggregated particles and incomplete crystallization, indicating limited atomic diffusion at this temperature. Upon increasing the temperature from 650°C to 750°C, a well‐defined, interconnected nanorod‐like nanostructure is observed. Here, the nanorod structure is uniformly distributed with reduced agglomeration, suggesting enhanced nucleation and controlled crystal growth (Figure S1). At 850°C, NbB_2_ exhibited dense nanoparticle packing and pronounced grain growth, leading to reduced morphological uniformity. Furthermore, at 950°C, NbB_2_ shows significant coarsening and severe agglomeration due to accelerated grain growth and sintering, which are detrimental to surface area exposure. Based on the initial screening, the sample prepared at 750°C is expected to perform best for the HER because it contains nanorods. The nanorod nanostructure can offer more active sites and a one‐dimensional (1D) conduction pathway, which can enhance overall electrochemical activity. Different magnifications of SEM images for optimized NbB_2_ and Se‐NbB_2_ are shown in Figure 1b–e. To optimize Se doping, the Se concentration was varied from 10 to 100 mg in hydrazine solution under MW radiation. Figure S2a–d shows the Se doping concentration variation SEM images of Se_x_‐NbB_2_ (x = 1, 3, 5, and 10 wt.% SeO_2_ relative to NbB_2_). No significant change in morphology is observed upon Se introduction; therefore, based on the electrochemical performance, we have optimized Se5‐NbB_2_ as the best electrode. The details of the electrochemical optimization for this have been discussed in the next section. As shown in Figure 1d,e, Se‐NbB_2_ retains a morphology like that of pristine NbB_2_, maintaining the interconnected nanorod‐like architecture without any noticeable structural change. This morphological stability can be attributed to the MW‐assisted doping mechanism, in which rapid volumetric heating induces localized high‐temperature regions via dipolar polarization and ionic conduction [49]. These localized effects facilitate the reduction of SeO_2_ and diffusion into the NbB_2_ surface without long‐term thermal treatment. The absence of significant morphological change after Se doping indicates that MW‐assisted Se incorporation primarily modifies the surface chemistry and electronic structure of NbB_2_ rather than altering its morphology [50]. Such preservation of the nanostructure, combined with electronic modulation and formation of new active sites, contributes to enhanced catalytic performance. The TEM images of NbB_2_ and Se‐NbB_2_ are shown in Figure 1f–h, clearly depicting the rod‐like nanostructure. The low‐magnification TEM image of NbB_2_ in Figure 1f (inset) shows a relatively uniform size distribution and is consistent with the SEM image. High‐resolution TEM (HR‐TEM) images of NbB_2_ in Figure 1i show lattice fringes with an interplanar spacing of 0.28 nm. Se‐NbB_2_ in Figure 1g exhibits a similar rod‐like morphology, suggesting Se doping does not alter the overall nanostructure. The selected area electron diffraction (SAED) pattern of Se‐NbB_2_ in Figure 1h (inset) shows distinct diffraction rings, confirming its polycrystalline nature while retaining structural integrity after doping. However, HR‐TEM analysis of Se‐NbB_2_ in Figure 1j reveals a lattice fringe spacing of approximately 0.33 nm, indicating lattice fringe expansion induced by Se incorporation. This increase in d‐spacing can be attributed to lattice distortion caused by the larger Se atoms in the NbB_2_ lattice. The observed lattice expansion and preserved crystallinity demonstrate that Se doping primarily modulates the local atomic arrangement and electronic structure of NbB_2_ without causing structural collapse, thereby enhancing catalytic activity.

**FIGURE 2:**
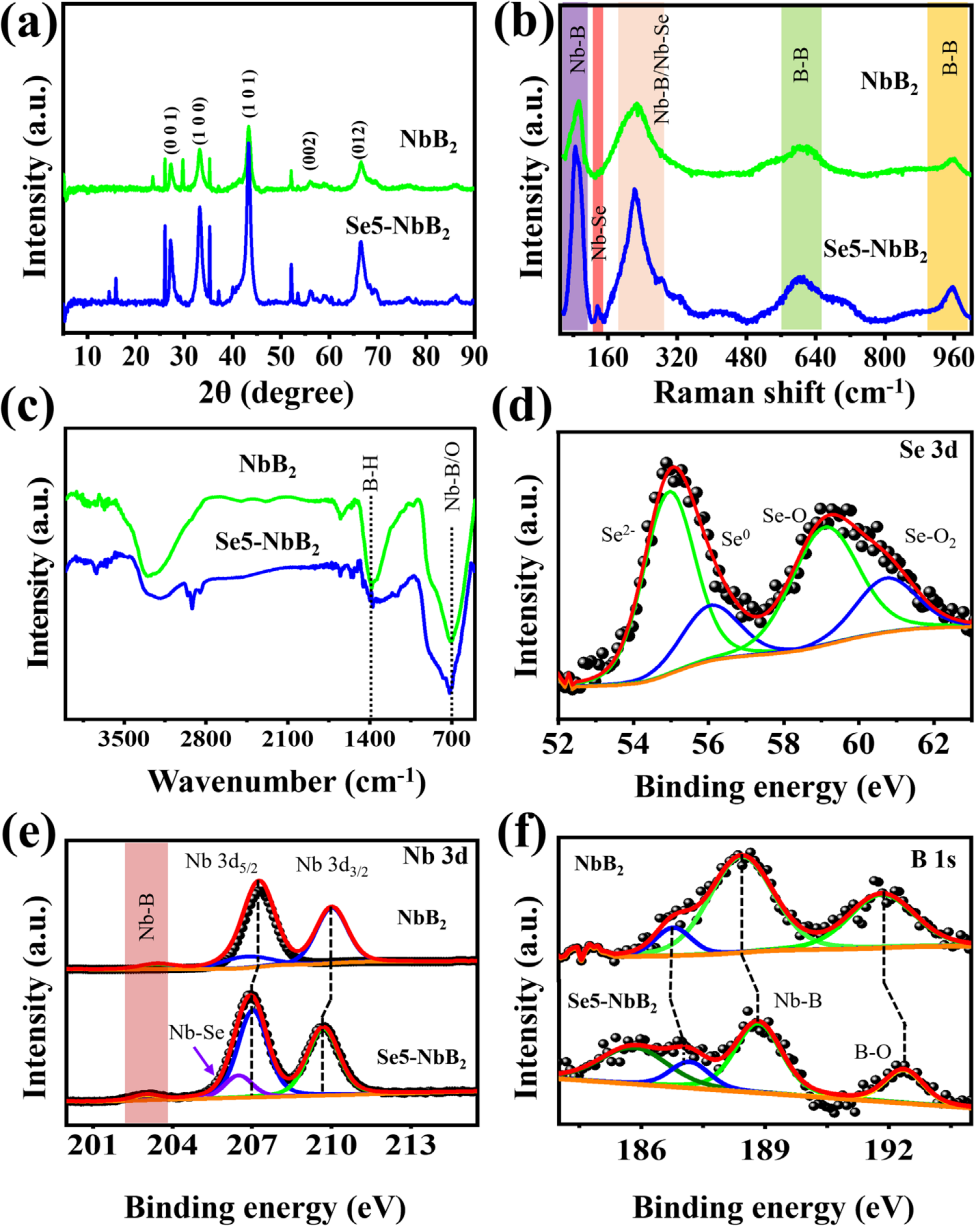
(a) XRD patterns, (b) Raman spectra, (c) FTIR, and high‐resolution XPS spectra of (d) Se 3d, (e) Nb 3d, (f) B 1s of pristine NbB_2_ and Se5‐NbB_2_.

As shown in Figure 1k, the energy‐dispersive X‐ray (EDX) elemental mapping confirms the presence of Se, Nb, and B within the Se5‐NbB_2_ samples, demonstrating a uniform distribution of all the elements. Brunauer–Emmett–Teller (BET) surface analysis was performed to evaluate the surfaces of pristine NbB_2_ and Se‐NbB_2_, as shown in Figure S3. Both samples exhibit typical nitrogen adsorption/desorption isotherms and H_4_ hysteresis behavior, confirming their porous nature. The BET surface area of the pristine NbB_2_ is calculated to be 12.25 m^2^ g^−^^1^, while Se5‐NbB_2_ shows an enhanced surface area of 14.76 m^2^ g^−^^1^, indicating that Se‐doping slightly enhances the accessible surface area. This increase in surface area is favorable for electrolyte diffusion and for exposing active sites, thereby enhancing the catalytic performance of Se‐NbB_2_. Figure 2a shows the XRD patterns of NbB_2_ and Se5‐NbB_2_, which exhibit the characteristic peaks of the NbB_2_ hexagonal phase (JCPDS No. 96‐151‐0779), confirming the successful formation of NbB_2_. The diffraction peaks observed at 2θ of 27.2, 33.1, 43.3, 56.0, and 66.5 can be indexed to (001), (100), (101), (002), and (012) crystallographic planes, respectively. The XRD spectra of NbB_2_ synthesized at different temperatures are shown in Figure S4a. After Se doping, the characteristic peaks remain unchanged. This indicates that Se incorporation does not alter the crystal structure of NbB_2_, preserving its phase integrity. The Raman spectra of pristine NbB_2_ and Se‐NbB_2_ are shown in Figure 2b, exhibiting characteristic vibrational features associated with their hexagonal AB_2_‐type structure. The Raman spectra at different temperatures are shown in Figure S4b. The peak positions in the low‐frequency region (around 80–200 cm^−^^1^) can be attributed to Nb−B lattice vibrations, while the broad band observed around 600–700 cm^−^^1^ corresponds to the E_2_g in‐plane stretching mode of B−B bonds within the boron layers [33]. A weak high‐frequency feature around 950–970 cm^−^^1^ can be assigned to second‐order or defect‐induced B−B vibrations. Upon incorporation of Se, additional peaks appear in the 100–250 cm^−^^1^ region, which are attributed to Nb−Se vibrational modes, confirming successful doping [51]. The noticeable shift and broadening of the B−B stretching mode, along with the enhanced intensity of the high‐frequency band, indicate lattice distortion, modification of the boron bonding environment, and increased defect density induced by Se incorporation [52]. These changes suggest that Se doping alters both the structural and electronic environment of NbB_2_, consistent with a defect‐assisted incorporation mechanism.

The FTIR spectra in Figure 2c show a broad band in the high wavenumber region (3200–3500 cm^−^^1^), corresponding to O−H stretching vibrations, indicating adsorbed moisture and surface hydroxyl groups. In the mid region (1400 cm^−^^1^), a distinct peak is assigned to B−H stretching [53]. In the low wavenumber region (600–800 cm^−^^1^), strong absorption bands are attributed to Nb−B and Nb−O/Nb−B−O lattice vibrations, reflecting the fundamental framework of NbB_2_. Upon Se incorporation, noticeable changes in peak intensity and broadening in the low‐wavenumber region indicate lattice distortion and modification of the local bonding environment. To further investigate more detailed electronic interactions between Se and NbB_2_ in the nanostructure, XPS was conducted on pristine NbB_2_ and Se‐NbB_2_. The full scan spectrum in Figure S5 reveals the presence of Nb, B, and O, and the presence of doped Se was confirmed by new peaks at 55 and 60 eV, suggesting that Se has successfully doped into NbB_2_. The presence of O may be due to surface adsorption of ambient contaminants, such as moisture.

The effect of Se doping on the electronic structure of NbB_2_ was investigated by fitting the high‐resolution spectra of Nb, B, and Se. The high‐resolution spectrum of Se 3d in Figure 2d can be deconvoluted into Se^2^^−^, Se^0^ (Se−Se), and oxidized Se species. The peak at 54.97 eV is assigned to Se^2^^−^, indicating the formation of Nb−Se bonds and confirming the successful incorporation of Se into the NbB_2_ lattice. The corresponding peak appears at a higher binding energy and overlaps with the Se^2^^−^ contribution. The peak around 56.06 eV is attributed to elemental Se (Se^0^), suggesting the presence of Se−Se bonding. Furthermore, the peaks at higher binding energies (59.13 and 60.74 eV) are associated with Se−O and Se−O_2_ species, which arise from surface oxidation of selenium upon exposure to air. Figure 2e shows the high‐resolution Nb 3d XPS spectra of pristine NbB_2_ and Se‐NbB_2_. Both NbB_2_ and Se‐NbB_2_ show two main prominent peaks around 207 and 210 eV associated with Nb 3d_5/2_ and Nd 3d_3/2_, respectively [54]. In addition, the small peak at 203.21 can be assigned to Nb−B. Notably, the existence of a new peak at 206.48 eV in the Se‐NbB_2_ spectrum suggests Se‐induced modification of NbB_2_. Moreover, the shift in the XPS spectra of Nb in the Se‐NbB_2_ and NbB_2_ can be attributed to the incorporation of Se into the NbB_2_, which indicates increased electron density around Nb atoms due to electronic interaction with Se. This electronic modulation weakens the Nb−H binding strength and optimizes hydrogen adsorption/desorption kinetics, which is highly favorable for the HER [55]. Figure 2f presents the high‐resolution B 1s XPS spectra.

For pristine NbB_2_, the deconvoluted B 1s spectrum shows characteristic peaks at 186.79 eV, 188.44, and 191.86 eV, which can be assigned to B−B, Nb−B, and B−O, respectively [56]. The XPS of B 1s in Se‐NbB_2_ exhibits a noticeable shift to higher binding energies, indicating a reduction in electron density around B atoms due to Se‐induced charge redistribution. Moreover, a new peak emerges at 185.78 eV, which can be attributed to a modified B−B environment, suggesting the formation of altered local bonding configurations in the boride lattice. Table S2 shows elemental composition (Atomic %) for the Se‐NbB_2_ samples from XPS analysis. As Se content increases, B content decreases, indicating successful Se doping with partial B‐site substitution and defect formation. Se has higher electronegativity than B, and then electron transfer can occur from Nb−B to Nb−Se, leading to charge redistribution and the generation of catalytically active sites. Such electronic and structural modulation is favorable for tuning hydrogen adsorption energies, thereby enhancing the HER activity of Se‐NbB_2_.

### 3.2 The Behavior of Se‐NbB_2_ as an Efficient Catalyst for HER

**FIGURE 3:**
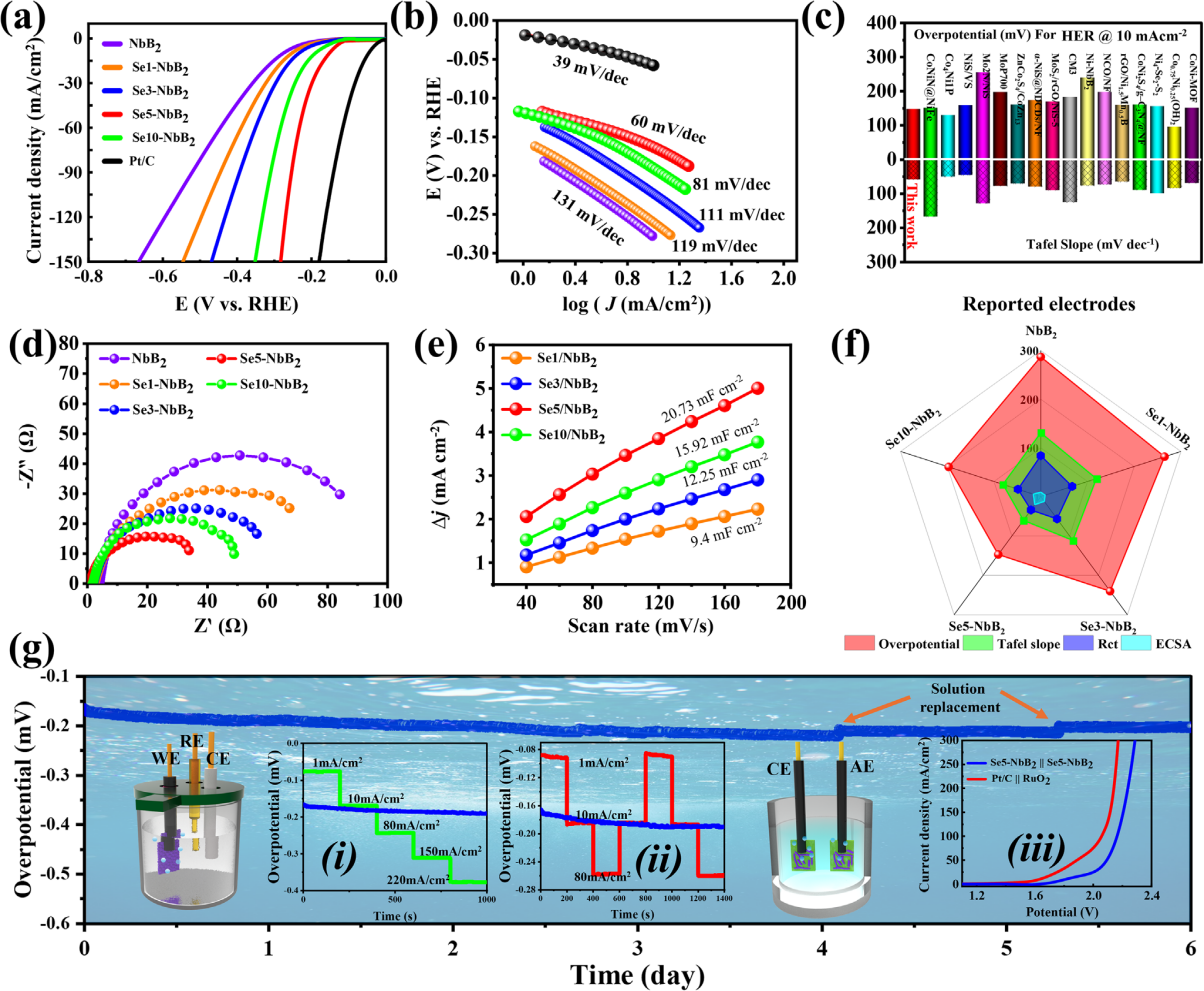
(a) HER LSV curves (b) corresponding Tafel slopes in 1 M KOH. (c) Comparison of HER and Tafel slopes of Se‐NbB_2_ with recent catalysts (References are reported in the supplementary). (d) Nyquist plots. (e) Calculated C_dl_ values. (f) Comparative spider chart of the HER catalytic performance. (g) Stability tests (inset (*i*) Chrono potential steps with different current densities, (*ii*) Chrono potential steps with current density switching (*iii*) LSV of the overall two‐system electrode).

To study the electrocatalytic activity of Se‐NbB_2_, we evaluated its HER performance under alkaline conditions. To fabricate the electrode, we coated Se‐NbB_2_ onto the carbon cloth as described in Section 2.5. The three electrode configurations were used to study HER performance, and the activity was further evaluated using a standard and 20 wt.% Pt/C as benchmarks. To optimize the synthesis conditions, HER performance was assessed under identical conditions for NbB_2_ prepared at different temperatures and with various Se doping levels. As illustrated in Figure S6, the HER performance of NbB_2_ prepared at 750°C was better than that of the others, as indicated by its lower overpotential than the other samples. As observed in the SEM and BET analyses, the sample prepared at 750°C shows a nanorod‐composed morphology, which provides more active sites for electrocatalytic activity compared to the other samples. Considering this, the sample prepared at 750°C was further used to optimize the Se concentration. To confirm the effect of Se doping on the HER performance of NbB_2_, different ratios of Se precursor to NbB_2_ were checked. LSV curves for HER performance at different ratios are shown in Figure 3a. The HER performance of NbB_2_ was improved by Se doping.

The nanostructured Se‐NbB_2_ prepared with 5% Se exhibits the best HER performance, delivering a low overpotential of 147 mV at a current density of 10 mA/cm^2^. This value is significantly lower than those of pristine NbB_2_ (292), Se1‐NbB_2_ (266 mV), Se3‐NbB_2_ (248 mV), and Se10‐NbB_2_ (203 mV) at 10 mA/cm^2^. At the same time, Se 5% in NbB_2_ achieves an optimal balance among electronic structure modulation, active site exposure, and charge transport, enabling favorable hydrogen adsorption/desorption energies and accelerating HER kinetics [57, 58]. At low Se levels, limited Se incorporation provides insufficient electronic modulation of the NbB_2_ lattice, resulting in poor charge transfer and hydrogen adsorption kinetics [59, 60]. Moreover, excessive Se doping (Se10‐NbB_2_) may lead to the formation of Se‐rich species, which can partially block Nb active sites and reduce electrical conductivity, thereby lowering catalytic performance [61, 62]. The corresponding Tafel slopes are shown in Figure 3b, emphasizing the optimization of catalytic reaction kinetics achieved by nanostructure engineering. The magnitude of the Tafel slope is closely related to the HER reaction rate. The smaller Tafel slope reflects enhanced charge‐transfer kinetics, demonstrating that the catalyst requires less overpotential to drive the HER at a desired current density. The Tafel slopes were calculated from the Tafel equation η = a + b log(j). The Se‐NbB_2_ exhibits a lower Tafel slope of 60 mV dec^−^^1^, indicating that Se doping accelerates HER kinetics via a Volmer–Heyrovsky mechanism, with the electrochemical desorption (Heyrovsky) step serving as the rate‐determining step [63, 64]. The Se5‐NbB_2_ catalyst exhibits superior electrochemical activity compared with recently reported non‐noble metal catalysts, as indicated in Figure 3c and Table S3. The unique molten‐salt‐assisted MW synthesis strategy demonstrates competitive HER electrocatalytic performance compared with conventional preparation methods, such as hydrothermal, solvothermal, and electrodeposition approaches, which are widely employed for the fabrication of transition‐metal‐based electrocatalysts. Se5‐NbB_2_ achieves lower overpotential (147 mV @10 mA cm^−2^), outperforming most reported cobalt‐, nickel‐, iron‐, molybdenum‐, vanadium‐, manganese‐, and zinc‐based electrocatalysts, indicating accelerated reaction kinetics and favorable charge‐transfer characteristics. Furthermore, its low Tafel slope (60 mV dec^−1^) indicates faster HER kinetics than many literature‐reported catalysts, demonstrating the effectiveness of Se5‐NbB_2_ in enhancing hydrogen evolution activity. To study charge transfer at the electrode surface, Nyquist plots were obtained and shown in Figure 3d. Se5‐NbB_2_ showed a lower charge transfer resistance (R_ct_) than the pristine NbB_2_ and all other fabricated electrodes, indicating faster charge transfer. The lower R_ct_ value suggests that Se doping acts as a promoter, facilitating charge transfer and enabling efficient H^+^ to H_2_ conversion. To determine the electrochemically active surface area (ECSA) of all electrodes, the non‐Faradaic region was selected between 0.2 and 0.3 V vs. RHE. As shown in Figure S7, cyclic voltammetry (CV) at different scan rates was used to determine the double‐layer capacitance (C_dl_), as shown in Figure 3e. The values of anodic and cathodic current densities are extracted from the CV curves at different scan rates and shown in Figure S7e. The calculated ECSA is shown in Figure S7f. Generally, a larger C_dl_ value shows higher electrochemically active sites, which can promote the H_2_ evolution efficiency. The Se5‐NbB_2_ electrode showed a higher ECSA of 518.25 cm^2^ than the other electrodes, indicating that 5% Se incorporation results in a greater number of exposed edges and active sites on the surface of the Se5‐NbB_2_ catalysts, which enhances HER performance. The comparative electrochemical performance metrics are summarized in the spider plot in Figure 3f. In comparison to other synthesized materials, Se5‐NbB_2_ exhibits superior overall electrochemical activity.

To further study the practical application of the electrode for water electrolysis, we applied Se‐NbB_2_ for long‐term stability via chronopotentiometry at fixed current densities (Movie S1). As shown in Figure 3g, chronopotentiometric measurements conducted in a three‐electrode system at a constant current density of 10 mA cm^−^^2^ over 6 days reveal that the Se‐NbB_2_ electrode maintains a highly stable response with minimal overpotential fluctuation, indicating outstanding long‐term durability. Moreover, electrode performance was evaluated at different current densities. As illustrated in Figure 3g (i), the electrode exhibits stable behavior even at higher current densities. The stability and reversibility of the catalyst were further assessed via chronopotentiometry under cyclic current density switching (Figure 3g(ii)). The current density was increased stepwise from 1 to 80 mA cm^−^^2^, then returned to 1 mA cm^−^^2^, followed by a second cycle. Notably, the overpotential corresponding to each current density remained nearly unchanged throughout the process, demonstrating excellent reversibility, fast charge transfer, and robust catalytic stability under varying operating conditions (Movie S2). Furthermore, to check the reproducibility of HER performance, three independently prepared Se5‐NbB_2_ electrodes were fabricated and tested under identical conditions, as shown in Figure S8. The LSV curves obtained from three independently prepared Se5‐NbB_2_ electrodes exhibited consistent HER activity, with an average overpotential of 149.7 mV at 10 mA cm^−2^ and a relative standard deviation (RSD) of 3.43%, demonstrating good reproducibility of the Se5‐NbB_2_ catalyst. In addition, LSV curves were recorded before and after the stability test, as shown in Figure S9, which shows negligible changes, confirming the excellent electrochemical durability of the Se5‐NbB_2_ catalyst. Overall, these results demonstrate that the optimized Se‐NbB_2_ catalyst exhibits outstanding HER activity, high reproducibility, and excellent long‐term stability.

On the other hand, the OER performance of the Se5‐NbB_2_ electrode was evaluated to explore its potential for overall water splitting. As shown in Figure S10, the electrode exhibits an overpotential of 420 mV at 10 mA cm^−^^2^ with a Tafel slope of 319 mV dec^−^^1^. Considering the promising bifunctional performance for both HER and OER, overall water splitting was investigated in a two‐electrode configuration (Figure 3g(iii)). The Se5‐NbB_2_||Se5‐NbB_2_ system requires a low cell voltage of 1.79 V to achieve 10 mA cm^−^^2^, which is competitive with previously reported electrocatalysts (Table S4).

### 3.3 HER Mechanism of Se‐NbB_2_ by In Situ Raman

**FIGURE 4:**
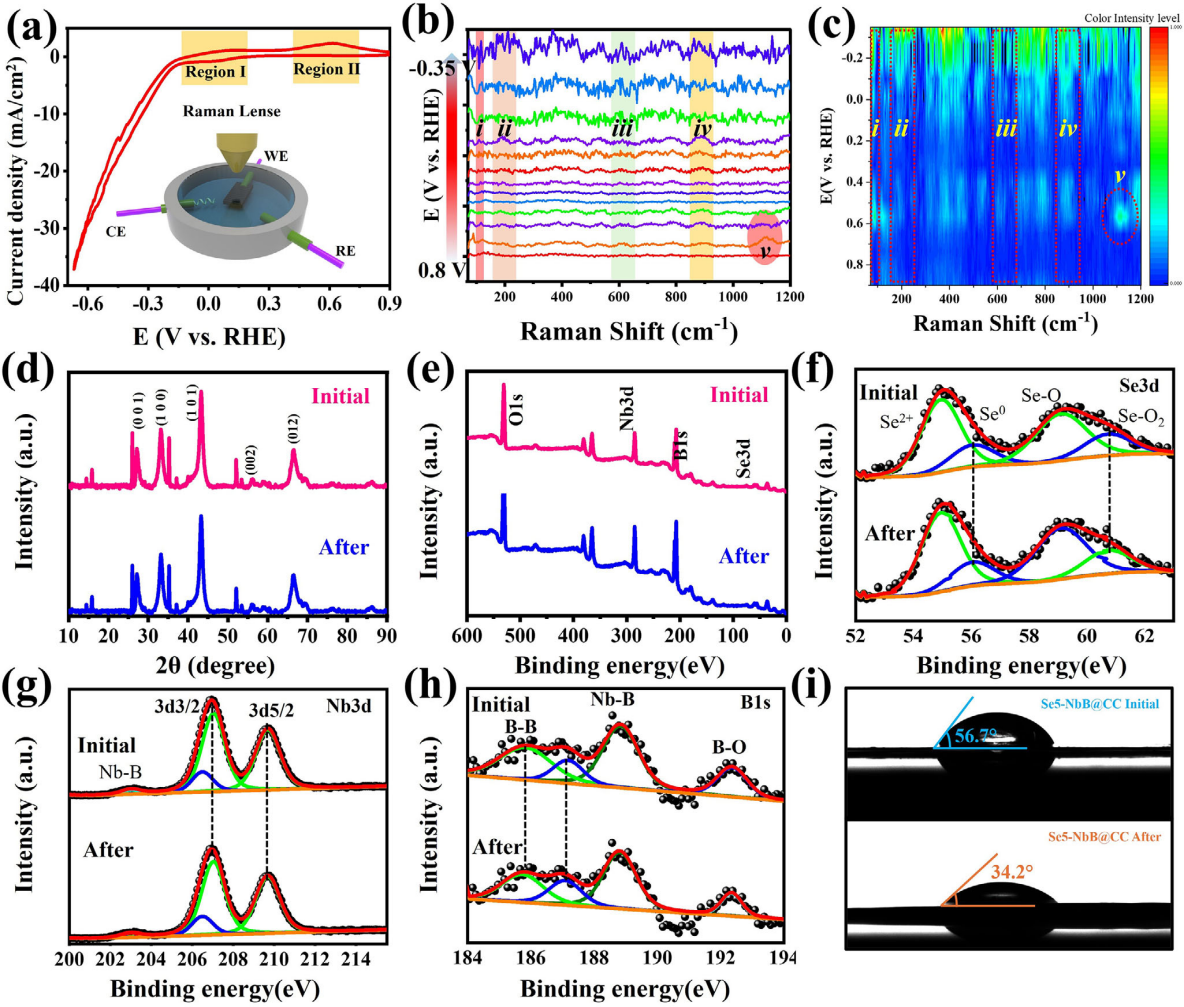
In situ Raman study. (a) CV curve (inset: in situ Raman assembly). (b) In‐situ Raman spectra of Se‐NbB_2_ tested at different potentials (vs. RHE) (c) Corresponding heat map picture. (d‐h) The initial and post‐HER structural study of Se‐NbB_2_ by (d) XRD (e) XPS spectra, and high‐resolution XPS spectra for (f) Se 3d (g) Nb 3d, and (h) B 1s. (i) Hydrophilicity testing of Se‐NbB_2_.

In‐situ Raman spectroscopy was employed to further elucidate the HER mechanism on the Se‐NbB_2_ catalyst under varying applied potentials. The assembly of the in‐situ Raman is shown in the inset picture of Figure 4a. The CV curve was performed in 1 M KOH electrolyte saturated with N_2_ in the assembly, which shows two distinct regions in the range of 0.9 to −0.65 V vs. RHE (Figure 4a). In the CV, region I (−0.1 to 0.2 V vs. RHE) shows one reversible peak, which may be due to interfacial adsorption/desorption processes involving H_2_O and OH^−^ species on Se‐NbB_2,_ which can be used for the HER reaction. Moreover, in region II (0.3 to 0.8 V vs. RHE), one oxidation peak appeared that may correspond to surface oxidation, likely associated with the formation of NbO_x_/SeO_x_ species under anodic potentials [65]. By confirming the CV behavior of the Se‐NbB_2_ in the system, we performed the Raman analysis while the selected potentials were fixed. The Raman spectra and corresponding heat mapping images of Se‐NbB_2_ from 0.8 to −0.35 V vs. RHE were plotted in Figure 4b,c. All the spectra were normalized for better comparison. The peak positions corresponding to NbSe (*i*), NbB/NbSe (*ii*), B−B (*iii*), and B−B (*iv*) remained consistent with those observed in the normal Raman spectra (Figure 2b). However, the intensities of all spectra were reduced compared to a normal Raman measurement, likely due to the presence of the electrolyte. A new peak appeared at approximately 1125 cm^−^^1^ upon applying 0.7 V vs. RHE, which may be attributed to the formation or oxidation of NbOx/SeOx species (Figure 4b,c). The Raman analysis did not show any significant changes during the change in potential up to 0.0 V vs RHE. However, as the potential reached the HER active region (below 0.0 V vs. RHE), new Raman features emerged and became more pronounced as overpotential increased. These features are attributed to the adsorption of water molecules and hydrogen intermediates on the catalyst surface during the Volmer and Heyrovsky steps in alkaline media. Specifically, Raman bands at 536, 785, 889, and 994 cm^−^^1^ reappear, indicating dynamic water adsorption/desorption processes on Se‐modified Nb active sites.

Further, ex‐situ XRD and XPS were performed to study the changes in the structure, composition, and morphology of the Se‐NbB_2_ catalyst after HER, as presented in Figure 4d‐i. The initial and post‐HER XRD spectra are shown in Figure 4d. This indicates that the characteristic peaks observed after the HER reaction match those of the initial XRD spectrum, suggesting no change in the Se‐NbB_2_ structural phase. XPS study was carried out to investigate possible surface chemical modifications of the catalyst after the HER reaction, as shown in Figure 4e–h. The characteristic XPS peaks of the Se‐NbB_2_ sample exhibit no noticeable changes before and after the HER, indicating that the overall surface composition and chemical states remain stable during the catalytic process. To check the wettability of the Se‐NbB_2_ for the HER application, the same amount of water was dropped on the electrode before and after HER performance. Figure 4i shows that the hydrophilicity of Se‐NbB_2_ is increased after HER due to electrochemical surface reconstruction during catalysis. As shown in Figure S11a,b, the SEM images obtained before and after HER reveal no noticeable change in the surface morphology, indicating excellent morphological stability of the Se5‐NbB_2_ catalyst. Furthermore, the HR‐TEM images in Figure S11c,d confirm that the nanorod morphology is well preserved after prolonged HER operation, with no significant change in the average nanorod dimensions (before HER 14.4 nm, after HER 14.6 nm). The difference is negligible, and no noticeable structural changes occur during electrocatalysis. Moreover, ICP‐OES analysis was performed before and after the HER operation (Table S5) to evaluate the elemental stability of Se5‐NbB_2_ and the possible Se leaching or catalyst reconstruction during electrocatalysis. The Se ratio remained unchanged, slightly changing from 5.41% to 5.40%, indicating negligible Se loss and excellent compositional stability during HER. Similarly, the Nb and B compositional ratios showed only negligible variations before and after HER, changing from 27.68% to 27.71% and 66.91% to 66.87%, respectively. Overall, the minimal changes in the Se, Nb, and B ratios demonstrate that the elemental composition of Se5‐NbB_2_ was well preserved during HER, with no significant Se leaching or catalyst reconstruction, confirming its excellent compositional stability and durability under alkaline HER conditions.

### 3.4 Electronic and Structural Modulation of NbB_2_ by Se Doping

**FIGURE 5:**
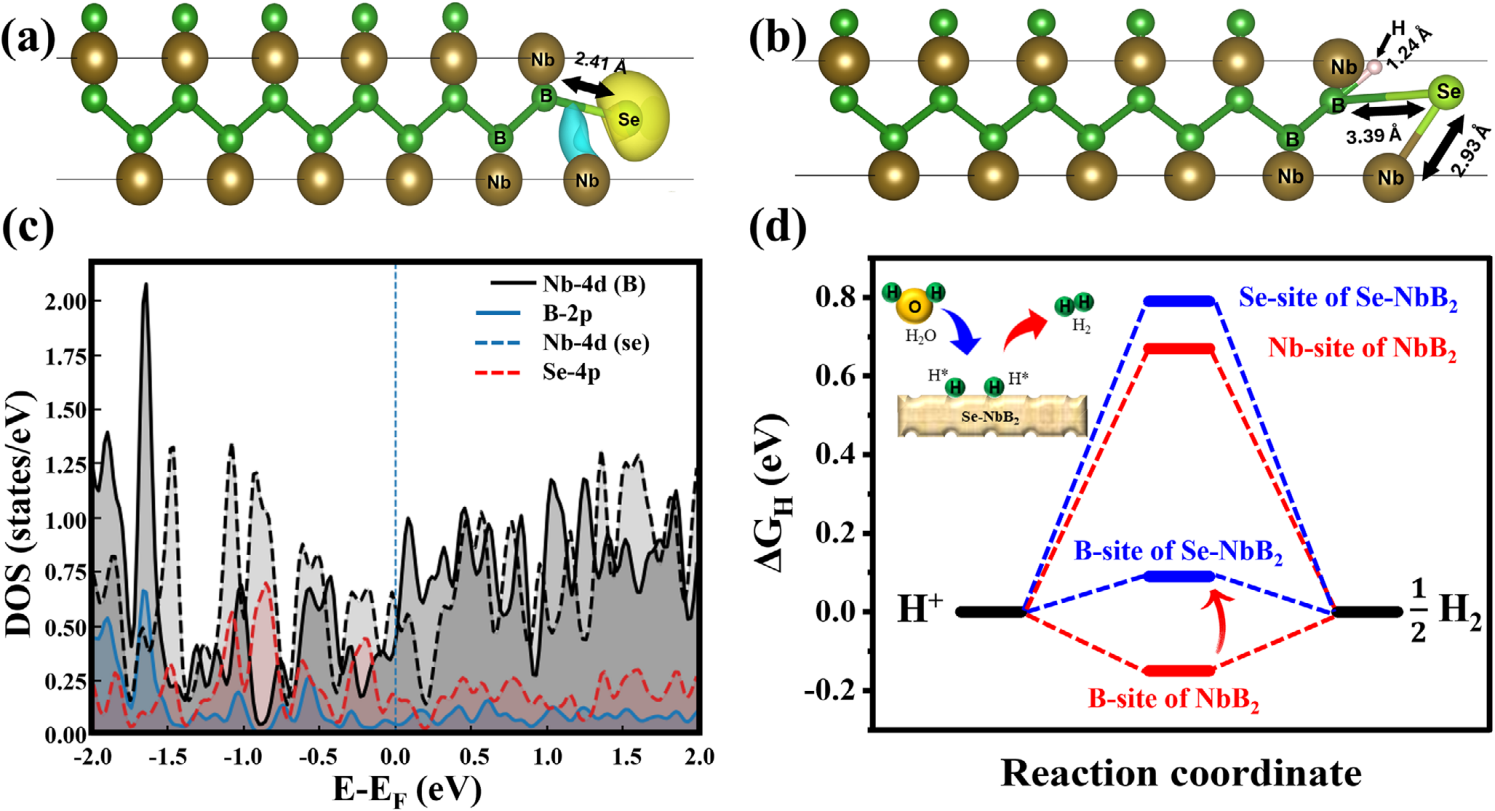
(a) Charge density difference of the NbB_2_ (101) surface with a single Se atom substituted at the boron site, referenced to the NbB_2_ surface. (b) Optimized atomic structure of a single hydrogen atom adsorbed at the boron site of the Se‐NbB_2_ (101) surface. (c) Projected density of states (PDOS) of Nb 4d, B 2p, and Se 4p orbitals for NbB_2_ and Se‐NbB_2_ (101) surfaces. (d) Gibbs free energy diagrams for hydrogen adsorption on NbB_2_ and Se‐NbB_2_ (101) surfaces.

Bulk NbB_2_ crystallizes in a high‐symmetry hexagonal structure (space group P6/mmm). To investigate its surface catalytic properties, a six‐layer NbB_2_ (101) slab model was constructed [66]. The bottom two layers were fixed to mimic the bulk environment, while the top four layers were fully relaxed. In the bulk‐like region, the Nb−B bond length remains nearly constant at 2.45 Å. However, upon approaching the surface, noticeable structural relaxation is observed, with bond lengths ranging from 2.41 to 2.49 Å, indicating surface‐induced lattice distortion. Substitutional B with Se induces significant local structural and electronic perturbations. The resulting B−Se bond length is 2.41 Å, whereas the Nb−Se bond extends to 2.93 Å, reflecting slight distortion due to the larger atomic radius of Se compared to B. Charge density difference analysis (Figure 5a) reveals pronounced electronic redistribution upon doping. At an isosurface value of 0.055 e, charge accumulation (yellow regions) is localized around the Se atom, accompanied by charge depletion (cyan regions) in its vicinity. This redistribution suggests strong electronic interaction between Se and Nb atoms. The modification in electronic structure is further supported by the density of states (DOS) (Figure 5c), where Se doping introduces a notable enhancement of p‐orbital contributions near the Fermi level (E_F_). This increase in electronic states near E_F_ indicates improved conductivity and enhanced charge transport characteristics.

The HER activity is evaluated by calculating the Gibbs free energy of hydrogen adsorption (ΔG_H_) to describe catalytic performance. The Gibbs free energy for hydrogen adsorption on various potential active sites of NbB_2_/Se‐NbB_2_ is calculated using the following equation:

(2) $$\Delta G_{H}=E_{ads}+\Delta ZPE-T\Delta S$$

where *E_ads_* is the adsorption energy of a hydrogen atom, Δ*ZPE* is the differential zero‐point energy (ZPE) between the adsorbed and gas phase of a hydrogen atom, −*T*Δ*S* is the entropic correction at room temperature. The ZPE of hydrogen in the gas phase (0.13 eV) and room‐temperature entropic corrections (0.20 eV) are taken from the NIST database [67]. The ZPE values of adsorbed hydrogen are calculated by adding the vibrational frequencies of the hydrogen atom adsorbed on the fixed surface. The adsorption energy of hydrogen on the boron site of pristine NbB_2_ is −0.42 eV. After incorporating differential zero‐point energy (ΔZPE = 0.07 eV) and entropic correction (‐TΔS), the resulting ΔG is −0.15 eV. Hydrogen adsorption on the niobium site is not energetically favorable, and hydrogen moves to the nearest boron atom. The calculated Gibbs free energy value for this case is 0.67 eV. Upon Se doping, the hydrogen adsorption energetics are significantly tuned toward thermoneutral conditions when hydrogen is adsorbed on the boron site. The Se‐doped NbB_2_ (101) surface with one hydrogen atom adsorbed at the surface B site is shown in Figure 5b. The E_ads_ weakens to −0.18 eV, while the ΔG is +0.09 eV for hydrogen adsorption at the B site adjacent to the Se dopant (Figure 5d). This value is remarkably close to the ideal thermoneutral condition (ΔG_H_ ≈ 0 eV), indicating an optimal balance between hydrogen adsorption and desorption, as predicted by the Sabatier principle. We also investigated the adsorption of hydrogen on the Se site, which is not favorable, leading to a high Gibbs free energy value of 0.79 eV (See Figure 5d).

The observed improvement in catalytic performance can be rationalized within the framework of the d‐band center theory. Electronic structure analysis reveals that Se substitution induces an upward shift in the Nb d‐band center. Specifically, the occupied d‐band center shifts from −2.69 to −2.47 eV. This shift arises from enhanced hybridization between Nb 4*d* and Se 4*p* orbitals, as can be seen in Figure 5c, which modifies the surface electronic structure. The upward movement of the d‐band center brings it closer to the Fermi level, reducing the occupation of antibonding states and thereby strengthening the interaction between surface Nb atoms and the adsorbed hydrogen atom. As a result, hydrogen adsorption is neither too strong nor too weak, leading to near‐optimal HER activity. Overall, Se doping effectively tailors both the geometric and electronic structure of the NbB_2_ (101) surface, transforming it into a highly efficient electrocatalyst for hydrogen evolution.

### 3.5 Triboelectric Nanogenerator Application of Se‐NbB_2_

Since Se incorporation substantially modifies the electronic structure of NbB_2_, the resulting changes in electrical conductivity and surface electronic states are expected to benefit both electrocatalytic hydrogen evolution and triboelectric energy harvesting. For HER, an efficient electrode requires rapid electron transport, favorable hydrogen adsorption, efficient interfacial charge transfer, and accessible catalytic sites. Incorporating Se into the NbB_2_ lattice induces electronic redistribution and modulates the electronic environment of the Nb–B framework, improving charge transport through the catalyst and facilitating electron transfer to the electrolyte/catalyst interface. These electronic modifications can promote more favorable hydrogen adsorption and accelerate the elementary HER reaction steps, contributing to the enhanced HER activity of Se–NbB_2_. In addition, the improved electrical conductivity minimizes resistive losses and facilitates rapid electron delivery to the catalytically active sites.

The same electronic characteristics are also advantageous for triboelectric energy harvesting. In a TENG, mechanical contact and separation generate triboelectric charges at the interface, while the electrode must efficiently collect and transport these charges to the external circuit. Thus, high electrical conductivity, efficient interfacial charge transfer, and favorable surface electronic properties are essential for minimizing charge‐transfer losses and improving charge collection. The Se‐induced electronic modulation in NbB_2_ provides an electrically conductive pathway for rapid extraction and transport of the generated triboelectric charges, thereby facilitating their effective collection by the electrode. Consequently, the electronic structure engineering achieved through Se incorporation simultaneously improves the charge‐transfer characteristics required for HER and the charge‐collection capability required for TENG operation, providing a common materials‐level origin for the enhanced performance in both applications.

**FIGURE 6:**
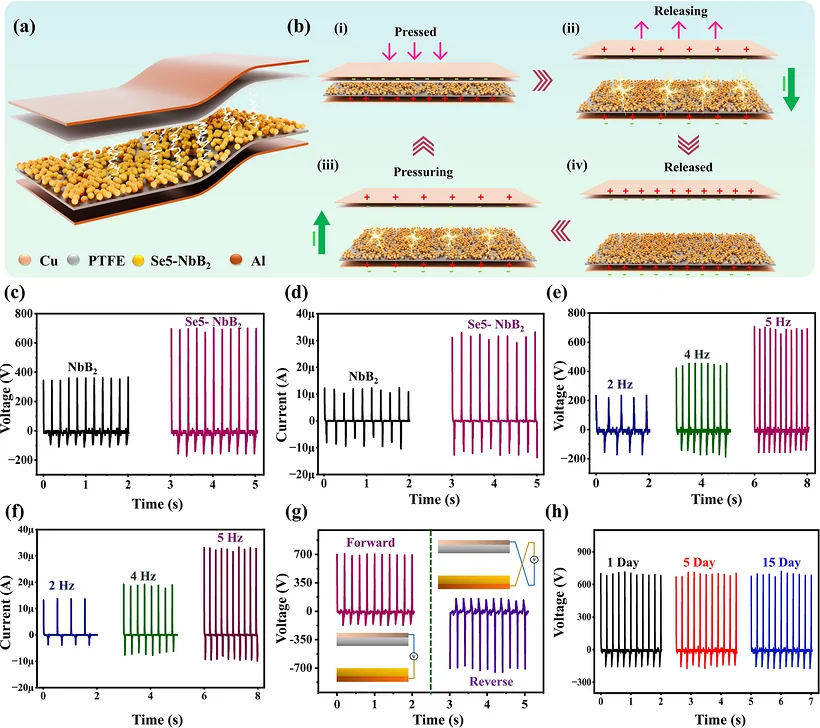
(a,b) Schematic illustration of the TENG device configuration and its operational principle; (c,d) Output voltage and current performance of TENG based on bare NbB_2_ and Se‐NbB_2;_ (e,f) Frequency‐dependent output voltage and current characteristics of the Se‐NbB_2_‐based TENG; (g) Polarity‐dependent output behavior of the TENG under forward and reverse configurations; (h) Stability and durability performance of the TENG device over different time intervals.

Recently, TENG has emerged as an effective technology for converting mechanical energy into electrical energy, offering significant potential for self‐powered systems and sustainable energy applications [9, 68, 69]. Its simple design, cost‐effectiveness, and ability to operate under low‐frequency mechanical stimuli make it highly suitable for next‐generation energy harvesting devices [70, 71]. In this study, a TENG device was fabricated as shown in Figure 6a, and its electrical output performance was systematically evaluated using bare NbB_2_ and Se‐NbB_2_ as active materials. To further understand the working principle of the fabricated device, the operating mechanism of the TENG was investigated under continuous mechanical stimulation in contact separation mode using a linear motor (NTI AG LinMot). Under compressive loading, the Se‐NbB_2_ and PTFE triboelectric layers established intimate contact, resulting in the formation of oppositely charged surfaces due to triboelectrification Figure 6b(i). Upon release of the applied force, the separation of these charged layers generated an electric potential difference across the electrodes, which drove electron flow from the bottom to the top electrode through the external circuit Figure 6b(ii). Once the layers were fully separated, the current ceased as the device reached electrostatic equilibrium Figure 6b(iii). Reapplication of the external force re‐established contact between the layers, reversed the induced electric field, and drove electrons in the opposite direction Figure 6b(iv). This cyclic contact separation process continuously generated an alternating electrical output signal. The voltage and current outputs were analyzed to understand the influence of material modification on charge generation and transfer behavior, as shown in Figure 6c,d.

The bare NbB_2_‐based TENG exhibited an output voltage of 359 V with a current of 13 µA, whereas the Se‐NbB_2_ device demonstrated an enhanced performance with a voltage of 703 V and a current of 33 µA. The significantly higher current output observed for the Se‐NbB_2_ device indicates improved electrical conductivity and more efficient charge transport resulting from the incorporation of Se. In addition, the increase in voltage output further supports the beneficial effect of doping on surface charge generation and transfer efficiency. Overall, these results confirm that Se doping plays a crucial role in enhancing the electrical performance of NbB_2_‐based TENG devices. To further investigate, the effect of operating frequency on the TENG output was investigated, as shown in Figure 6e,f, revealing that both voltage and current increased with increasing frequency. This behavior is attributed to the higher rate of contact separation cycles, which results in greater charge generation per unit time. In the Se‐NbB_2_‐based TENG, the voltage increased from 234 to 703 V as the frequency varied from 2 to 5 Hz, while the current output increased from 13 to 33 mA. The Se‐NbB_2_ device demonstrated a more pronounced response to frequency variation, suggesting enhanced capability to accommodate rapid charge transfer processes under dynamic mechanical conditions. To further elucidate the performance of the device, the power output was analyzed as a function of the external load resistance. To systematically evaluate the device behavior, the load resistance was varied over a wide range from 100 to 100 MΩ. As shown in Figure S12, an increase in load resistance resulted in a corresponding decrease in output current. Nevertheless, the power output increased with resistance up to an optimal value, owing to the trade‐off between current and voltage. The device achieved a maximum instantaneous power of 1968 µW at a load resistance of 30 MΩ, indicating the optimal operating condition for efficient energy harvesting. Furthermore, the polarity‐dependent behavior of the TENG was examined under forward and reverse configurations to evaluate directional charge transport, as shown in Figure 6g. The forward condition exhibited higher current output, whereas the reverse condition showed lower current, indicating asymmetric charge transfer behavior and effective rectification. This confirms that the generated electrical signals can be directionally controlled under different operational conditions. To evaluate long term performance, the durability and stability of the TENG device were assessed over 1, 5, and 15 days, as shown in Figure 6h. The initial measurements showed maximum output performance, followed by a slight decrease over time, which may be attributed to minor surface changes or repeated mechanical operation. Nevertheless, the device retained a substantial portion of its electrical output even after 15 days, demonstrating good stability. The Se‐NbB_2_‐based TENG exhibited better performance retention than the bare NbB_2_, indicating improved material robustness and long‐term operational reliability. Furthermore, TENGs represent a promising technology for harvesting mechanical energy into usable electrical power for battery‐free systems. The practical applicability of the Se‐NbB_2_/PTFE based TENG was demonstrated by successfully operating commercial LEDs and a calculator under simple manual tapping, as shown in Movies S3 and S4, highlighting its potential for real‐world self‐powered electronic applications. Overall, these results demonstrate that Se doping significantly enhances the electrical output and stability of the Se‐NbB_2_ TENG device, highlighting the importance of material engineering and electrical conductivity in optimizing triboelectric performance. Se incorporation improves the electronic properties and charge‐transfer characteristics of NbB_2_, which also contribute to its enhanced HER activity; the superior TENG performance is consistent with the same Se‐induced electronic modulation.

## 4 Conclusion

In summary, NbB_2_ electrodes were successfully produced via a simple molten salt method, followed by MW‐assisted Se doping. The sample prepared at 750°C with 5% Se doping showed excellent electrocatalytic activity for HER and excellent voltage/current output for the TENG, demonstrating the effectiveness. The Se‐NbB_2_ catalyst provides efficient, durable HER activity in 1 M KOH, attributed to its high ECSA, low charge transfer resistance, and redistributed charge density. In‐situ Raman spectroscopy indicates that water molecules are readily adsorbed on the catalyst at HER relevant potentials, thereby aiding the Volmer‐Heyrovsky pathways. The catalytic activity of Se‐NbB_2_ toward the HER demonstrates a low overpotential of 147 mV and durability over six days in three electrode systems. The full‐cell water‐splitting system with Se‐NbB_2_ reached 10 mA cm^−2^ at 1.79 V. Overall, the Se doping modifies the structural and electronic properties of the NbB_2_ (101) surface, resulting in nearly thermoneutral H_2_ adsorption. This optimized ΔG_H_, driven by shifts in the d‐band center, enhances HER catalytic activity. Importantly, the same material also demonstrates strong performance in TENG, producing high voltage and current outputs. This dual functionality highlights Se‐NbB_2_ as a versatile platform that bridges energy conversion and harvesting. Overall, this work establishes chalcogen doping as an effective strategy to regulate boride catalysts and provides a unique design principle for developing multifunctional, noble metal‐free materials for integrated energy systems.

## Author Contributions

**Shalmali R Burse**: writing – original draft, validation, investigation, formal analysis, data curation. **Vikram Mahamiya**: software, formal analysis. **Ali Mohammadi**: software, formal analysis. **Swapnil R Patil**: software, formal analysis. **Harshitha B Tyagaraj**: formal analysis. **Gagankumar S k**: formal analysis. **Amal Alghafri**: funding acquisition, supervision, resources. **Ebrahim Al Hajri**: funding acquisition, resources, supervision. **Nilesh R. Chodankar**: conceptualization, supervision, writing – review and editing, resources, funding acquisition. **Yun Suk Huh**: funding acquisition, writing – review and editing, supervision, resources. **Young Kyu Han**: funding acquisition, resources, supervision.

## Conflicts of Interest

The authors declare no conflicts of interest.

## Supporting information




**Supporting File 1**: advs77902‐sup‐0001‐SuppMat.docx.

**Supporting File 2**: advs77902‐sup‐0002‐SuppMat.pptx.

## Data Availability

The data that support the findings of this study are available from the corresponding author upon reasonable request.
