# Supplementary material for: Tailoring Electronic Structure of NbB2 Nanorods Through Selenium Incorporation for Synergistically Enhanced Hydrogen Evolution and Triboelectric Energy Harvesting
Source: Adv Sci (Weinh). 2026 Sep 27:e77902. Online ahead of print. doi: 10.1002/advs.77902 (PMC13616364; doi:10.1002/advs.77902)
Supplement: Supplementary file 1 [file ADVS-9999-e77902-s002.docx]

Supporting Information

**Tailoring Electronic Structure of NbB_2_ Nanorods Through Selenium Incorporation for Synergistically Enhanced Hydrogen Evolution and Triboelectric Energy Harvesting**

*Shalmali R Burse^a^, Vikram Mahamiya^b,c^, Ali Mohammadi^a^, Swapnil R Patil^d^, Harshitha B Tyagaraj^a^,* *Gagankumar S K^a^, Amal AlGhafri^e^, Ebrahim Al Hajri^f^, Nilesh R. Chodankar^f^*, Yun Suk Huh^d^*, Young Kyu Han^a^**

^a^Department of Energy and Material Engineering, Dongguk University, Seoul, 04620, Republic of Korea

^b^Center for Interdisciplinary Research, SRM University-AP, Amaravati, Andhra Pradesh 522240, India

^c^Department of Physics, SRM University-AP, Amaravati, Andhra Pradesh 522240, India.

^d^Department of Biological Sciences and Bioengineering Nanobio High-Tech Materials Research Center, Inha University, Incheon 22212, Republic of Korea

^e^Rabdan Academy, 114646, Abu Dhabi, United Arab Emirates

^f^Mechanical and Nuclear Engineering Department, Khalifa University of Science and Technology, Abu Dhabi, 127788, United Arab Emirates

*Corresponding authors: [nilesh.chodankar@ku.ac.ae](mailto:nilesh.chodankar@ku.ac.ae), [yunsuk.huh@inha.ac.kr](mailto:yunsuk.huh@inha.ac.kr), [ykenergy@dongguk.edu](mailto:ykenergy@dongguk.edu).

**Figure. S1.** HR-SEM images of NbB_2_ synthesis temperature optimization. (a) 650 °C, (b) 750 °C, (c) 850 °C, (d) 950 °C.

**Figure. S2.** HR-SEM images of Se doping concentration variation. (a) Se1-NbB_2_, (b) Se3-NbB_2_, (c) Se5-NbB_2_, (d) Se10-NbB_2_.

**Figure. S3.** Nitrogen adsorption/desorption isotherm spectra of NbB_2_ and Se5-NbB_2_.

**Figure. S4.** (a) XRD patterns and (b) Raman spectra of NbB_2_ synthesized at different temperatures.

**Figure. S5.** XPS survey spectra for NbB_2_ and Se5-NbB_2_.

**Figure. S6.** Electrocatalytic HER and OER performance of NbB_2_ catalyst synthesized at different temperatures.

**Figure. S7.** (a-d) Cyclic voltammetry curves of (a) Se1-NbB_2_ (b) Se3-NbB_2_ (c) Se5-NbB_2_ (d) Se10-NbB_2_ at scan rates from 20 to 160 mV s^-1^. (e) Anodic and cathodic current densities extracted from the CV curves. (f) Double-layer capacitance (C_dl_) values calculated from the linear fitting of anodic and cathodic currents.

**Figure. S8.** Repeated HER LSV curve for Se5-NbB_2_ on three different electrodes.

**Figure. S9.** HER LSV curve before and after stability test.

**Figure. S10.** OER LSV curve of Se-NbB_2_ with different Se doping concentrations.

**Figure. S11.** (a-b) SEM images, (c-d) HR-TEM images before and after HER performance.

**Figure. S12.** Instantaneous power of the Se-NbB_2_ TENG.

**Table S1.** Comparison of recently reported heteroatom-modified boride electrocatalysts with the present Se-NbB_2_ catalyst.

| Electrode | Dopant | Doping method | Required time | Temperature in °C | Refs. |
| --- | --- | --- | --- | --- | --- |
| Se-NbB_2_ | Se | Microwave | 2 min | 100-110 | This work |
| P-NCB | P | Tube furnace | 30 min | 350 | ^[1]^ |
| S-ReB_2_ | S | Tube furnace | 1 hour | 1000 | ^[2]^ |
| CC@N-CoP | N | Tube furnace | 2.5 hours | 300 | ^[3]^ |
| F-Co_2_B | F | pyrolysis | 2 hours | 600 | ^[4]^ |
| FVB-2 | V | Chemical reduction | 4 hours | - | ^[5]^ |
| La-MoBx | La | Molten salt | 4 hours | 800 | ^[6]^ |
| B-Ni_3_Fe | B | exothermic reaction/ carbonization | 2 hours | 400-600 | ^[7]^ |
| B-CoSe_2_/CC | B | Hydrothermal Gas-phase selenization | 6 hours | 120 | ^[8]^ |
|  | Se |  | 1 hour | 450 |  |

**Table S2.** XPS elemental composition (At.%) for NbB_2_ and Se-NbB_2_ samples.

| Samples | Nb | Se | B |
| --- | --- | --- | --- |
| NbB_2_ | 27.97 | - | 72.03 |
| Se1-NbB_2_ | 28.34 | 0.92 | 70.74 |
| Se3-NbB_2_ | 29.12 | 3.27 | 67.61 |
| Se5-NbB_2_ | 27.68 | 5.41 | 66.91 |
| Se10-NbB_2_ | 28.57 | 9.63 | 61.80 |

**Table S3.** Comparison table for HER performance of non-noble metals electrodes in alkaline electrolyte and corresponding Tafel slopes.

| Electrode | Synthesis Methods | Overpotential @10 mA/cm^2^ | Tafel slope mV dec^-1^ | Refs. |
| --- | --- | --- | --- | --- |
| Se5-NbB_2_ | Molten salt/MW | 147 | 60 | This work |
| CoNiN@NiFe LDH | Electrodeposition | 150 | 169 | ^[9]^ |
| Co_4_Ni_1_P | Solvothermal | 129 | 52 | ^[10]^ |
| NiS/VS | Hydrothermal | 158 | 47.2 | ^[11]^ |
| Mo_2_N/NiS-5:1 | Hydrothermal | 254 | 130.08 | ^[12]^ |
| MoP_700_ | Chemical reduction/ phosphidation | 196 | 79 | ^[13]^ |
| ZnCo_2_S_4_/CoZn_13_ | Hydrothermal | 160 | 72.28 | ^[14]^ |
| α-NiS@NDCDs/NF | Hydrothermal | 173 | 81 | ^[15]^ |
| MoS_2_/rGO/NiS-5 | Hydrothermal | 169 | 91.6 | ^[16]^ |
| CM3 | Electrodeposition | 182 | 127 | ^[17]^ |
| Ni-NbB_2_ | Molten salt | 239 | 77.72 | ^[18]^ |
| 3NCO/NF | Hydrothermal | 196 | 75 | ^[19]^ |
| rGO/Ni_1.5_Mn_0.5_B | Hydrothermal | 159 | 67 | ^[20]^ |
| CoNi_2_S_4_/g–C_3_N_4_@NF | Hydrothermal | 160 | 90.8 | ^[21]^ |
| Ni_4_-Se_2_-S_2_ | Hydrothermal | 155 | 101 | ^[22]^ |
| Co_0.75_Ni_0.25_(OH)_2_ | Hydrothermal | 95 | 85 | ^[23]^ |
| CoNi-MOF | Solvothermal | 150 | 71 | ^[24]^ |

**Table S4.** Comparison table for overall water splitting performances.

| Electrode | Voltage @ 10 mA/cm^2^ | Reference |
| --- | --- | --- |
| Se5-NbB_2_ | 1.79 | This work |
| NBU-4/NF@CoOOH | 1.65 | ^[25]^ |
| CoNiN@NiFe LDH | 1.63 | ^[9]^ |
| La_0.025_-Mo_0.975_P@N/C | 1.73 | ^[26]^ |
| CoMo@CN | 1.74 | ^[27]^ |
| Ni-MMT | 1.82 | ^[28]^ |

**Table S5.** The ICP data before and after HER performance of Se5-NbB_2_

| Elements | Se5-NbB_2_ before HER | | Se5-NbB_2_ after HER | |
| --- | --- | --- | --- | --- |
|  | Concentration (ppm) | percentage | Concentration (ppm) | percentage |
| Se | 54.15 | 5.41 | 53.77 | 5.40 |
| Nb | 277.00 | 27.68 | 275.56 | 27.71 |
| B | 669.52 | 66.91 | 664.86 | 66.87 |
|  | | | | |

**References**

1. A. T. Sivagurunathan, S. Seenivasan, T. Kavinkumar, and D.-H. Kim, “Phosphorus Doping of Nickel–Cobalt Boride to Produce a Metal–Metalloid–Nonmetal Electrocatalyst for Improved Overall Water Splitting,” *Journal of Materials Chemistry A* *12*, no. 8 (2024): 4643–4655, https://doi.org/10.1039/D3TA07382C.

2. Y. Wu, Y. Sun, B. Li, et al., “Molten Salt-Assisted Synthesis of S Anion Doped Rhenium Diboride Nanosheets for Efficient Hydrogen Evolution in Wide PH Range,” *Journal of Alloys and Compounds* *1040* (2025): 183617, https://doi.org/https://doi.org/10.1016/j.jallcom.2025.183617.

3. Q. Zhou, Z. Shen, C. Zhu, et al., “Nitrogen-Doped CoP Electrocatalysts for Coupled Hydrogen Evolution and Sulfur Generation with Low Energy Consumption,” *Advanced Materials* *30*, no. 27 (2018): 1800140, https://doi.org/https://doi.org/10.1002/adma.201800140.

4. H. Han, H. Choi, S. Mhin, et al., “Advantageous Crystalline–Amorphous Phase Boundary for Enhanced Electrochemical Water Oxidation,” *Energy & Environmental Science* *12*, no. 8 (2019): 2443–2454, https://doi.org/10.1039/c9ee00950g.

5. T. Ahmed, S. Pal, N. Sinha, C. Das, and P. Roy, “Amorphous Vanadium-Doped Iron Borate/Tetraboride Hybrid as an Efficient Electrocatalyst for the Oxygen Evolution Reaction,” *Dalton Transactions* *55*, no. 12 (2026): 4932–4940, https://doi.org/10.1039/d5dt03026a.

6. L. Zhang, X. Wang, H. Ye, et al., “Rare-Earth Lanthanum Doped Molybdenum Borides for Electrocatalytic Hydrogen Evolution Reaction,” *Journal of Alloys and Compounds* *1022* (2025): 179876, https://doi.org/https://doi.org/10.1016/j.jallcom.2025.179876.

7. Y. B. Adegbemiga, N. Ullah, M. Xie, et al., “Ni3Fe Nanoparticles Enclosed by B-Doped Carbon for Efficient Bifunctional Performances of Oxygen and Hydrogen Evolution Reactions,” *Journal of Alloys and Compounds* *835* (2020): 155267, https://doi.org/10.1016/j.jallcom.2020.155267.

8. Z. Liu, D. Gao, L. Hu, et al., “Boron-Doped CoSe2 Nanowires as High-Efficient Electrocatalyst for Hydrogen Evolution Reaction,” *Colloids and Surfaces A: Physicochemical and Engineering Aspects* *646* (2022): 128903, https://doi.org/10.1016/j.colsurfa.2022.128903.

9. J. Wang, G. Lv, and C. Wang, “A Highly Efficient and Robust Hybrid Structure of CoNiN@NiFe LDH for Overall Water Splitting by Accelerating Hydrogen Evolution Kinetics on NiFe LDH,” *Applied Surface Science* *570* (2021): 151182, https://doi.org/https://doi.org/10.1016/j.apsusc.2021.151182.

10. L. Yan, L. Cao, P. Dai, et al., “Metal‐organic Frameworks Derived Nanotube of Nickel–Cobalt Bimetal Phosphides as Highly Efficient Electrocatalysts for Overall Water Splitting,” *Advanced Functional Materials* *27*, no. 40 (2017): 1703455.

11. K. Bao, Y. Yan, T. Liu, T. Xu, J. Cao, and J. Qi, “Constructing NiS–VS Heterostructured Nanosheets for Efficient Overall Water Splitting,” *Inorganic Chemistry Frontiers* *7*, no. 24 (2020): 4924–4929.

12. Y. Zang, S. Huang, B. Yang, G. Chen, X. Liu, and N. Zhang, “Constructing Collaborative Interface between Mo2N and NiS as Efficient Bifunctional Electrocatalysts for Overall Water Splitting,” *Applied Surface Science* *611* (2023): 155656, https://doi.org/https://doi.org/10.1016/j.apsusc.2022.155656.

13. X. Xie, M. Song, L. Wang, et al., “Electrocatalytic Hydrogen Evolution in Neutral PH Solutions: Dual-Phase Synergy,” *Acs Catalysis* *9*, no. 9 (2019): 8712–8718.

14. D. Zhao, M. Dai, H. Liu, Z. Duan, X. Tan, and X. Wu, “Bifunctional ZnCo2S4@CoZn13 Hybrid Electrocatalysts for High Efficient Overall Water Splitting,” *Journal of Energy Chemistry* *69* (2022): 292–300, https://doi.org/https://doi.org/10.1016/j.jechem.2022.01.042.

15. C. Pitchai, S. M. Gopalakrishnan, and C.-M. Chen, “Ultra-Efficient Nitrogen-Doped Carbon Dots-Supported Nickel Sulfide as a Platinum-Free Electrocatalyst for Overall Water Splitting in Basic Medium,” *Energy & Fuels* *38*, no. 3 (2024): 2235–2247.

16. G. Liu, K. Thummavichai, X. Lv, et al., “Defect-Rich Heterogeneous MoS2/RGO/NiS Nanocomposite for Efficient PH-Universal Hydrogen Evolution,” *Nanomaterials* *11*, no. 3 (2021): 662, https://doi.org/10.3390/nano11030662.

17. J. Reddy, M. Duraivel, K. Senthil, and K. Prabakar, “Kinetically Controlled Mn Doping Effect on Composition Tuned CoMn Hydroxide Nanoflakes for Overall Water Splitting Application,” *ChemCatChem* *14*, no. 22 (2022): e202200966.

18. Y. Sun, B. Li, J. Lang, G. Che, Y. Wu, and M. Shang, “Electronic Tailoring of Ni–NbB 2 Nanorods via Molten Salt Synthesis for High-Efficiency Alkaline Hydrogen Evolution,” *New Journal of Chemistry* *49*, no. 38 (2025): 16828–16834, https://doi.org/10.1039/D5NJ02968F.

19. S. Rathod, K. K. Joshi, P. M. Pataniya, C. K. Sumesh, and S. Kapatel, “Nb Doping Strategy for Active Site Modification in Co3O4to Enable Concurrent Hydrogen Production and Glycerol Valorization for Efficient Formate Production,” *Journal of Materials Chemistry A* (2026): 4268–4281, https://doi.org/10.1039/d5ta08434b.

20. S. R. Burse, S. H. Talib, H. B. Tyagaraj, et al., “Precious-Metal-Free RGO/NiMnB Nanoarchitectonics with Covalent Metal Support Interaction for Efficient and Durable Alkaline Water Splitting,” *Nano Convergence* *12*, no. 1 (2025): 50.

21. R. Zahra, E. Pervaiz, M. M. Baig, and O. Rabi, “Three-Dimensional Hierarchical Flowers-like Cobalt-Nickel Sulfide Constructed on Graphitic Carbon Nitride: Bifunctional Non-Noble Electrocatalyst for Overall Water Splitting,” *Electrochimica Acta* *418* (2022): 140346, https://doi.org/https://doi.org/10.1016/j.electacta.2022.140346.

22. Y. Li, H. Du, Y. Su, et al., “Construction of Heterostructured NiS/NiSe2 and Their Application in Electrocatalytic Water Splitting,” *International Journal of Hydrogen Energy* *66* (2024): 286–293, https://doi.org/https://doi.org/10.1016/j.ijhydene.2024.04.101.

23. X. Wang, Z. Li, D. Wu, et al., “Porous Cobalt–Nickel Hydroxide Nanosheets with Active Cobalt Ions for Overall Water Splitting,” *Small* *15*, no. 8 (2019): 1804832.

24. S. K. Konavarapu, G. Kim, K. Shin, and S. Y. Kim, “Boosting Electrocatalytic Activity of Bimetallic CoNi‐MOF for OER and HER Through a Synergistic Bimetallic Approach,” *Chemistry–A European Journal* *31*, no. 24 (2025): e202500010.

25. N. Diyali, S. Diyali, M. Chettri, et al., “Operando Electro-Oxidation Reconstitution of a Newly Designed 2D Co(II)-MOF on Nickel Foam to Cobalt Oxyhydroxide Nanosheets towards Energy Sustainability: A Holy Grail in Electrocatalytic Water Splitting,” *ACS Applied Materials & Interfaces* *17*, no. 6 (2025): 9351–9363, https://doi.org/10.1021/acsami.4c20152.

26. X. Ye, H. Ma, S. Wu, et al., “Electron Structure Customization of Molybdenum Phosphide via Lanthanum Doping toward Highly Efficient Overall Water Splitting,” *Journal of Materials Science and Technology* *218* (2025): 227–235, https://doi.org/10.1016/j.jmst.2024.08.043.

27. J. Jiang, Q. Liu, C. Zeng, and L. Ai, “Cobalt/Molybdenum Carbide@ N-Doped Carbon as a Bifunctional Electrocatalyst for Hydrogen and Oxygen Evolution Reactions,” *Journal of Materials Chemistry A* *5*, no. 32 (2017): 16929–16935.

28. N. Patel, B. Saini, S. Ponnada, R. Singhal, and R. K. Sharma, “N/S‐Codoped Nickel Oxide Supported on Activated Montmorillonite Clay as an Efficient Bifunctional Electrocatalyst for Overall Water Splitting,” *European Journal of Inorganic Chemistry* *28*, no. 4 (2025): e202400603.
